# The management of asymptomatic carotid stenosis: Is there a benefit to operate elderly patients?

**DOI:** 10.37825/2239-9747.1017

**Published:** 2020-10-01

**Authors:** NM Bouayed, A Sekkal

**Affiliations:** 1 University hospital Hai Sabah 31000 - Oran Algeria

**Keywords:** carotid stenosis, elderly population, carotid angioplasty, carotid endarterectomy, stroke

## Abstract

**Background:**

We present a retrospective study of a series of 40 patients over the age of 75 operated for an asymptomatic carotid stenosis. The results were evaluated during an average of 3 years of follow-up.

**Material and method:**

The study is retrospective and monocentric. The series includes 40 patients aged over 75 years and with an average age of 78.5 years (range 75–82). Patients underwent surgery for an asymptomatic carotid stenosis of more than 80%. The technique in all case was a carotid endarterectomy.

**Results:**

There have been no postoperative deaths or neurological adverse events. During an average follow-up of 3 years, there was one death secondary to colon cancer. However, 5 patients were lost to follow-up.

**Conclusion:**

Carotid surgery in elderly patients may have a benefit. However, our study has shortcomings. It is retrospective and the patient cohort is reduced. A randomized, prospective study, comparing surgery or angioplasty with the best medical treatment, is necessary to choose the most effective and safest treatment to offer to an elderly patient with asymptomatic carotid stenosis.

## I. INTRODUCTION

Stroke is the leading cause of non-traumatic acquired disability. 15–20% of strokes are linked to atheromatous carotid stenosis. The prevalence of carotid stenosis increases with age, especially after age 75 and especially among men.

Carotid stenosis are asymptomatic if no hemispheric or retinal symptoms have appeared in the 6 months preceding the discovery of the stenosis. Asymptomatic carotid stenosis (ACS) accounts for about ¾ of all carotid lesions. The goal of treating carotid stenosis is to prevent a stroke. While carotid surgery is recommended in the elderly patients in cases of symptomatic carotid stenosis, that of ACS in patients over 75 years old, even when tight, remains subject to many controversies. Indeed, the debate, concerning the benefit to be expected from a surgical procedure or a possible angioplasty procedure, is not yet closed. The aim of our study, through a retrospective evaluation of the results of carotid endarterectomy (CEA) of a series of 40 patients aged over 75 years, is to answer the question of the usefulness or not, of operate the tight ACS affecting the elderly population.

## II. MATERIAL AND METHOD

We performed a retrospective monocentric analysis of a series of 40 patients with an ACS of more than 80%. All the patients were more than 75 years old and had a life expectancy of more than 4 years. This series includes 28 men and 12 women. The mean age was 78.5 years (range 75–82).

ACS was constantly discovered during the assessment of atheromatous disease. High blood pressure was present in 60% of patients, diabetes in 35% of cases, a history of smoking in 50% of cases and dyslipidemia in 40% of cases.

Based on the risk score described by Wallaert [1], we estimated that 34 patients (85%) could have a life expectancy of more than 5 years and 6 patients (15%), a life expectancy of at least four years. Duplex ultrasound, performed in all cases, allowed the measurement of the degree of stenosis and the evaluation of the characteristics of the atherosclerotic plaque.

The main criterion for inclusion of patients in the series is a degree of stenosis greater than 80% measured by the maximum systolic velocity at the site of the stenosis which must have been greater than 250 cm / s.

The second inclusion criterion was the ultrasound characteristics of a possibly dangerous and unstable plaque. CT angiography or angio-MRI of the brain and supra-aortic trunks was requested for all patients. All our patients were operated under general anesthesia. The operative technique consisted of an open thromboendarterectomy in 34 patients, 15 of whom were closed with a patch and 19 without patch. One patient was treated with a homolateral carotid-carotid bypass.

## III. RESULTS

The mean clamping time was 22 minutes. The average hospital stay was 4 days (range 3–7). 3 patients had a minor cervical hematoma. 02 patients had regressive cognitive impairment in 2 days.

There were no neurological events or deaths during the first 30 post-operative days. During an average follow-up of 3 years (range 0.5 and 5), 5 patients were lost to follow-up. One patient died, 4 years later, secondary to a colon cancer and another patient suffered from a state of dementia. The rest of the patients followed had no ischemic stroke.

## IV. DISCUSSION

In some countries, such as Denmark, an ACS is very little operated, while in other countries like the United States, the majority of the carotid stenosis operated, is asymptomatic [2]. Indeed, some authors believe that revascularization is not a solution in ACS, all ages combined [3].

The results of our study are generally good and encouraging. However, the study has flaws: it is retrospective, the cohort is small, the number of people lost to follow-up is relatively large and finally, no comparison with a group of patients, treated with best medical treatment, was carried out. These shyness mean that the conclusions of our study could not lead us to specify the therapeutic indications to be adopted in real life with respect to ACS in elderly patients. Thus, in our institution we refer to published randomized studies and to the various recommendations of learned societies for the choice of the best therapeutic option to offer to our elderly patients with ACS.

An older person is more likely to have a stroke than a young subject. An older person’s stroke is more disabling and with a higher risk of death than that which occurs in a young subject [4]. However, research of ACS is not routinely recommended in the elderly [5]. It should be noted that prospective randomized studies have often excluded patients over the age of 75 with ACS. Indeed, age has always been considered a surgical risk factor. However, at the end of our study, we noted that age did not increase the operative risk of carotid surgery. This observation was also underlined in the NASCET study, but in patients with symptomatic carotid stenosis [6]. A meta-analysis published by Ballota, collecting 845 CEA performed in patients over 75 years old but symptomatic, found an average cumulative post-operative mortality and neurological morbidity rate of 3.3% [7]. Another confirmation comes from a Canadian study which shows that carotid surgery in elderly patients, even asymptomatic, is not accompanied by an increase in mortality and neurological morbidity but that the post-operative complications, particularly myocardial infarction, should consider a reflection on the advisability of performing CEA in an elderly patient [8].

Thus, the question of the best treatment arises with more reflection when the carotid stenosis is asymptomatic. Optimal medical treatment, including correction of risk factors, one or two antiplatelet agents, a statin and controlled physical activity, has significantly reduced the risk of stroke in patients with ACS. The annual rate of stroke of ACS in patients on optimal medical treatment is currently around 0.5% [2]. The ACST-1 study, which compared a group of patients treated with CEA to another group treated medically, did not find a benefit in terms of prevention of stroke, in patients over 75 years old with ACS [9]. So, what elderly patient can benefit from a carotid interventional procedure? We believe, that under certain conditions, an elderly patient should benefit from carotid revascularization if his stenosis is very tight, by more than 80%, or if it progresses rapidly, if his life expectancy exceeds 4 years, if he is no cognitive impairment, if the plaque ultrasound structure raises the risk of an embolic risk (irregular or echolucent plaque) or if the MRI shows an intra-plaque hemorrhage, or if the transcranial doppler reveals transient signals of high intensity from spontaneous micro-embolization or if a silent ipsilateral cerebral infarction is found or a decrease in vascular reactivity [9]. If the therapeutic indication is selected, what type of anesthesia will be made for the realization of carotid revascularization? Locoregional anesthesia is often recommended to operate on elderly patients, but in our series, all of our patients had general anesthesia without this being harmful. A final question is that of the choice of technique: endarterectomy or carotid angioplasty-stenting( CAS)? Stanziale compared carotid angioplasty in patients over the age of 80 to those under the age of 80 and concluded that the risk of angioplasty is much higher in the elderly than in the young [10] Results, which contradict Stanziale’s conclusion, are reported by another study that compared a group of 129 patients treated with CAS to another group of 45 patients treated with CEA. All patients were over 80 years of age. 80% of patients were asymptomatic. There were 2.3% death and stroke at 30 days in the group treated by CAS and 4.4% in the group treated by CEA. The author of this study underlines that if the CAS is carried out by an experienced team, octogenarians can benefit from it with more safety [11]. The complex arterial anatomy of the elderly may explain the high rate of complications after CAS in patients over 80 years of age [12].

In order to reduce the rate of neurological complications after CAS, trans-carotid angioplasty has developed and has become safer and more effective in octogenarians, whether symptomatic or asymptomatic, as pointed out in an article published by Ghamraoui which found that the clamping procedure and time were short and that the hospital stay was also short. [13]. However, whatever the technique chosen, open surgery or angioplasty-stenting, it must be performed by a trained team which has an activity whose combined mortality and neurological mortality rate is less than 3%. [14].

## V. CONCLUSION

The individual risk of stroke in ACS is very low, even in cases of high stenosis, and the absolute benefit of surgery in these cases is very low. Patients were, theoretically, more surgical risk factors than young patients. However, on condition of making a rigorous selection, a patient over 75 years old, with few well-controlled comorbidities and a good life expectancy, can benefit from an interventional procedure in case of tight ACS. The decision will be made on a case-by-case basis by a multidisciplinary team. Patient information is essential. We believe that the safest technique for an elderly patient is trans-carotid angioplasty. However, in order to confirm this therapeutic option, a randomized prospective study on a large series is necessary.

## References

[1] Wallaert JB, Cronenwett JL, Bertges DJ (2013). Optimal selection of asymptomatic patients for carotid endarterectomy based on predicted 5-year survival. J Vasc Surg.

[2] Venermo M, Wang G, Sedrakyan A (2017). Carotid Stenosis Treatment: Variation in International Practice Patterns, European Journal of Vascular and Endovascular Surgery. Eur J Vas Endovasc Surg.

[3] Hart RG, Ng KH (2015). Stroke prevention in asymptomatic carotid artery disease: revascularization of carotid stenosis is not the solution. Pol Arch Med Wewn.

[4] ECST European Carotid Surgery Trialists’Collaborative Group (1998). Randomised trial of endarterectomy for recently symptomatic carotid stenosis: final results of the MRC European Carotid Surgery Trial (ECST). Lancet.

[5] Naylor AR (2013). Asymptomatic carotid artery stenosis : State of the art management. J Cardiovasc Surg (Torino).

[6] Alamovitch S, Eliasziw M, Algra A, Meldrum H, Barnett HJM, NASCET, for the North American Symptomatic Carotid Endarterectomy Trial (NASCET) Group (2001). Risk, causes, and prevention of ischaemic stroke in elderly patients with symptomatic internal-carotid-artery stenosis. Lancet.

[7] Ballota E, Da Giau G, Saladini M, Abbruzzese E (1999). Endartériectomie carotidienne chez les malades symptomatiques ou non âgés de 75 ans et plus: mortalité et morbidité neurologique péri-opératoire. Ann Chir Vasc.

[8] Doonan RJ, Abdullah A, Steinmetz-Wood S, Corriveau MM, Mackenzie KS, Gill HL (2019). Carotid Endarterectomy Outcomes in the Elderly: A Canadian Institutional Experience.

[9] Halliday A, Harrison M, Hayter E (2010). 10-year stroke prevention after successful carotid endarterectomy for asymptomatic stenosis (ACST-1): A multicentre randomised trial. Lancet.

[10] Stanziale FS, Marone LK, Boules TN (2006). Carotid artery stenting in octogenarians is associated with increased adverse outcomes. J Vasc Surg.

[11] Fantozzi C, Taurino M, Rizzo L, Stella N, Persiani F (2016). Carotid Endarterectomy or Stenting in Octogenarians in a Monocentric Experience.

[12] Lin SC, Trocciola SM, Rhee J, McKinsey JF, Kent KC, Faries PL Analysis of Anatomic Factors and Age in Patients Undergoing Carotid Angioplasty and Stenting.

[13] Ghamraoui AK, Ricotta JJ (2020). Outcomes Of Transcarotid Artery Revascularization (TCAR). Octogenarians and Older.

[14] Naylor AR, Ricco JB, de Borst GJ, Writing Group (2018). Management of atherosclerotic carotid and vertebral artery disease: 2017 Clinical Practice Guidelines of the European Society for Vascular Surgery (ESVS). Eur J Vasc Endovasc Surg.

